# Challenges in Recycling Spent Lithium‐Ion Batteries: Spotlight on Polyvinylidene Fluoride Removal

**DOI:** 10.1002/gch2.202200237

**Published:** 2023-02-05

**Authors:** Mengmeng Wang, Kang Liu, Jiadong Yu, Qiaozhi Zhang, Yuying Zhang, Marjorie Valix, Daniel C.W. Tsang

**Affiliations:** ^1^ Department of Civil and Environmental Engineering The Hong Kong Polytechnic University Hung Hom Kowloon Hong Kong China; ^2^ Research Centre for Environmental Technology and Management The Hong Kong Polytechnic University Hung Hom Kowloon Hong Kong China; ^3^ State Key Joint Laboratory of Environment Simulation and Pollution Control School of Environment Tsinghua University Beijing 100084 China; ^4^ School of Chemical and Biomolecular Engineering University of Sydney Darlington NSW 2008 Australia

**Keywords:** cathode materials, circular economy, EV battery recycling, lithium recovery, sustainable waste management

## Abstract

In the recycling of retired lithium‐ion batteries (LIBs), the cathode materials containing valuable metals should be first separated from the current collector aluminum foil to decrease the difficulty and complexity in the subsequent metal extraction. However, strong the binding force of organic binder polyvinylidene fluoride (PVDF) prevents effective separation of cathode materials and Al foil, thus affecting metal recycling. This paper reviews the composition, property, function, and binding mechanism of PVDF, and elaborates on the separation technologies of cathode material and Al foil (e.g., physical separation, solid‐phase thermochemistry, solution chemistry, and solvent chemistry) as well as the corresponding reaction behavior and transformation mechanisms of PVDF. Due to the characteristic variation of the reaction systems, the dissolution, swelling, melting, and degradation processes and mechanisms of PVDF exhibit considerable differences, posing new challenges to efficient recycling of spent LIBs worldwide. It is critical to separate cathode materials and Al foil and recycle PVDF to reduce environmental risks from the recovery of retired LIBs resources. Developing fluorine‐free alternative materials and solid‐state electrolytes is a potential way to mitigate PVDF pollution in the recycling of spent LIBs in the EV era.

## Graphical abstract

The state‐of‐the‐art separation technologies are evaluated for cathode materials and Al foil of spent lithium‐ion batteries, including physical separation, solid‐phase thermochemistry, solution chemistry, and solvent chemistry, as well as the corresponding reaction behavior and transformation mechanisms of the organic binder polyvinylidene fluoride.

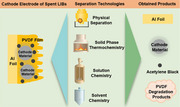

## 1 Introduction

Global emissions of greenhouse gases including carbon dioxide, methane, nitrous oxide, and fluorinated gases from use of fossil fuels have increased significantly since the 1900s and today these contribute to over three‐quarters of total emissions.^[^ ^1^, ^2^ ^]^ Electric vehicles (EVs) are an important part of the mix of low‐carbon energy sources that are urgently needed to mitigate global climate change.^[^ ^3^ ^]^ Although the trend towards the use of EVs can facilitate the reduction of greenhouse gases, there are concerns on the impact of EVs production on the ecosystems and its potential human toxicity.^[^ ^4^ ^]^ These impacts should be alleviated through circular economy approaches that require efficient recycling of retired battery materials.^[^ ^5^, ^6^ ^]^ The recycling technologies for EVs batteries, however, are still in their infancy, which may pose a challenge in achieving circularity goals.

The use of EVs is increasing rapidly worldwide. According to International Energy Agency (IEA), the global EV sales in 2021 were 6.6 million, which was double from the production in the previous year.^[^ ^7^ ^]^ The IEA predicts that by 2030 there will be over 145 million EVs in use and most if not all will be powered by lithium‐ion batteries (LIBs). Considering that the service life of the battery pack is usually 6–8 years, globally this will usher in a wave of end‐of‐life EVs.^[^ ^8^, ^9^ ^]^ The challenges that will be posed by the corresponding generation of huge numbers of end‐of‐life EVs and LIBs to the environmental protection are still unknown, but is it expected to be significant unless appropriate recycling technologies are developed. The LIBs contain toxic but valuable non‐renewable metals such as lithium (Li), nickel (Ni), cobalt (Co), manganese (Mn), aluminum (Al), and copper (Cu), as well as fluorine compounds such as lithium hexafluorophosphate (LiPF_6_) and polyvinylidene fluoride (PVDF).^[^ ^10^ ^]^ If not properly recycled, end‐of‐life LIBs would pose health, ecological, and hazardous risks in addition to substantial wastage of valuable resources.^[^ ^11^, ^12^ ^]^ The pollution control of organic compounds in solid waste has always been a grand challenge.^[^ ^13^, ^14^, ^15^ ^]^

**Figure 1:**
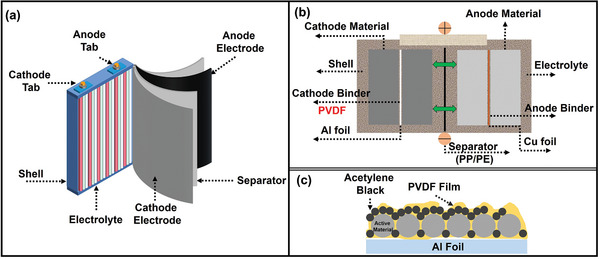
Structure diagrams of LIBs. a) Main components. Reproduced with permission.^[^ ^17^ ^]^ Copyright 2018, Elsevier. b) Internal structure. Reproduced with permission.^[^ ^21^ ^]^ Copyright 2022, John Wiley and Sons. c) Structure of cathode materials and Al foil (Yellow coating represents PVDF film). Reproduced with permission.^[^ ^23^ ^]^ Copyright 2019, American Chemical Society.

Part of the challenges in recycling LIBs include the cost and the complexity of chemical features and structures that make it difficult to disassemble.^[^ ^16^ ^]^ The LIBs mainly include five components, i.e., shell, cathode electrode, anode electrode, separator, and organic electrolyte (**Figure** **1**a).^[^ ^17^ ^]^ The Cu foil is usually used as the substrate of the anode electrode, and the anode electrode material is evenly coated on both sides. The anode material comprises anode active material (e.g., graphite, silicon, and carbon) and cathode binder (e.g., styrene‐butadiene rubber, acrylic resin, and sodium carboxymethyl cellulose). Because the anode binder is generally a water‐soluble chemical, Cu foil can be separated from anode active material via ultrasonic treatment combined with water immersion.^[^ ^18^, ^19^ ^]^ Al foil is usually used as the substrate of the cathode electrode, and the cathode material is evenly coated on both sides.^[^ ^20^ ^]^ The cathode material includes the cathode active material, conductive agent acetylene black, and organic binder PVDF (Figure 1b).^[^ ^21^, ^22^ ^]^ The cathode active material and conductive agent acetylene black are chemically bonded with current collector Al foil by PVDF (Figure 1c).^[^ ^23^ ^]^

The cathode active materials in LIBs are divided into lithium cobaltate (LiCoO_2_, LCO), lithium iron phosphate (LiFePO_4_, LFP), lithium manganite (LiMnO_2_, LMO), and ternary nickel cobalt manganese (LiNi*_x_* Co*_y_* Mn_1‐_ *_x‐y_* O_2_, NCM).^[^ ^24^, ^25^ ^]^ The main economic driver for recycling the retired LIBs is the recovery of valuable metals from cathode materials.^[^ ^26^ ^]^ The physical and chemical properties of valuable metals in the form of polymetallic compounds are relatively stable with lower environmental impact; their recycling is principally driven by resource utilization.^[^ ^27^, ^28^ ^]^ In contrast, the fluorine‐containing compounds in the LIBs show a higher environmental risk, especially the LiPF_6_ and PVDF.^[^ ^25^, ^29^ ^]^ The International Agency for Research on Cancer (IARC) of the World Health Organization published a list of carcinogens in 2017, three of which include fluorine.^[^ ^30^, ^31^ ^]^ Nevertheless, there are few studies of recycling organic fluorides from the retired LIBs because of their low content and economic value when compared to the valuable metals of LIBs.^[^ ^32^ ^]^ Thus, comprehensive research and discussion in the holistic recycling of end‐of‐life LIBs are incredibly urgent.

Because of different chemical nature and structures of LIBs components, recycling of each part will require various technologies. An important step in recycling valuable metals from LIBs is the efficient separation of the battery components specifically the cathode material, PVDF and the Al foil.^[^ ^33^, ^34^ ^]^ The complete separation of the cathode will ensure all the Al foil is recovered to allow its recycling by smelting. Complete removal of the PVDF will reduce potential fouling by the remaining organic film on cathode materials that could hamper the leaching efficiency of the metallic components by hydrometallurgy. In addition, the removal of Al foil will avoid its leaching and re‐precipitation during the hydrometallurgical step simplifying the metal extraction step. Finally, recovery and recycling of the organic binder PVDF from LIBs will avoid its potential environmental pollution and promote green recycling of LIBs.^[^ ^35^, ^36^ ^]^ The holistic recycling is an important step in improving the environmental performance of retired LIBs.

To date, very few studies have specifically addressed the decomposition and recovery of PVDF in the field of spent LIBs recycling.^[^ ^35^ ^]^ As an organic fluorine product, PVDF of the spent LIBs can easily enter the environmental matrices through crushing, pyrolysis, incineration, and thermochemistry, thereby affecting the surrounding environment and human receptors.^[^ ^37^, ^38^ ^]^ Monitoring the migration, transformation, and pollution control of PVDF during the recycling of spent LIBs will be a global challenge. To provide useful information for recycling and pollution control of retired LIBs, this review articulates the separation technologies of cathode materials and Al foil, as well as the migration and transformation mechanisms of PVDF in different processing technologies. This review aims to provide technical guidance for the global recycling community on spent LIBs, particularly concerning the fate of PVDF.

## 2 Composition and Binding Mechanism of PVDF

### 2.1 Composition and Structure

**Figure 2:**
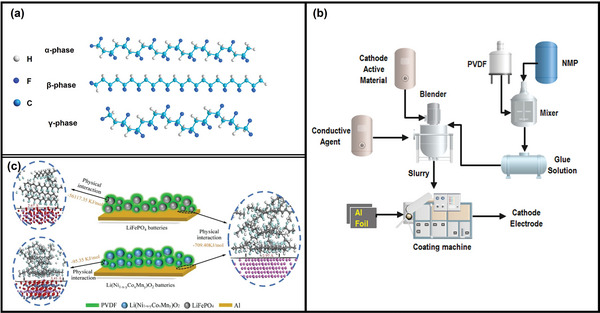
a) Different crystal types of PVDF α, β, and γ. Reproduced under the terms of the open access Creative Common CC BY license.^[^ ^41^ ^]^ Copyright 2019, Molecular Diversity Preservation International (MDPI). b) Application process of PVDF. Reproduced with permission.^[^ ^44^ ^]^ Copyright 2022, Elsevier. c) Bonding mechanism of PVDF in different LIBs. Reproduced with permission.^[^ ^53^ ^]^ Copyright 2021, Elsevier.

Because of advantageous features including good thermal stability, high viscosity, and easy film formation, PVDF (CH_2_‐CF_2_ *_n_*) has been the most commonly used organic binder in LIBs.^[^ ^39^, ^40^, ^41^ ^]^ Its melting point is 170 °C, and the thermal decomposition temperature is about 350 °C. PVDF is a white powder‐like polymer with a crystallinity of ≈50%. Five crystal types of PVDF (i.e., α, β, γ, δ, and ε) have been reported so far, and α, β and γ are the most common crystal types (**Figure** **2**a).^[^ ^41^ ^]^ These crystal types can convert to each other under certain conditions (e.g., heat, electric field, machine, and radiation energy). PVDF is characterized by high mechanical strength and good corrosion resistance. With a good chemical stability, PVDF is not easily corroded by acid, alkali, strong oxidants, and halogens at room temperature.^[^ ^42^, ^43^ ^]^ Only a few chemicals, such as fuming sulphuric acid, strong alkali, and ketone, can induce swelling or partial dissolution. During the fabrication process of LIBs, the PVDF is first dissolved to colloid solution by strongly polar organic solvents such as *n*‐methylpyrrolidone (NMP), dimethylacetamide, and dimethyl sulphoxide (Figure 2b).^[^ ^44^ ^]^ Subsequently, the colloid solution is evenly mixed with the slurry and conductive agent of cathode materials, and then they are coated on the current collector Al foil. After the volatilization of organic solvent, the solidified PVDF becomes an effective medium for binding cathode materials to Al foil.^[^ ^45^ ^]^

### 2.2 PVDF Functions

As the organic binder, only a small quantity of PVDF is used in the LIBs production, accounting for 1.4‐2.5% of the cost of LIBs, but it is irreplaceable.^[^ ^46^, ^47^ ^]^ The functions of PVDF are as follows: 1) ensure the uniformity and safety of active materials for pulping; 2) bind the active materials among themselves; 3) bind the active materials to the current collector; 4) facilitate the solid electrolyte interface formation on graphite surface; and 5) improve the cycle performance, fast charge and discharge, and reduce the internal resistance of LIBs.^[^ ^48^, ^49^ ^]^ Therefore, the PVDF not only binds the cathode material particles to Al foil, but it also enhances the electrical contact between cathode material and conductive agent as well as between cathode material and current collector, thus stabilizing the electrode structures. Simultaneously, the PVDF can buffer the expansion and shrinkage of the anode and cathode in LIBs. As a result, the PVDF plays an indispensable role in LIBs development. However, its strong bonding and high chemical stability can make disassembly difficult. Traces of PVDF in the cathode electrode will result in pollution of the residual organic fluoride in the recycling process, compromising the environmental performance of LIBs.^[^ ^50^, ^51^ ^]^

### 2.3 Mechanism of Bonding

Evaluation and understanding the bonding between PVDF and cathode material/Al foil is an important step in efficient recycling of retired LIBs,^[^ ^52^ ^]^ which can be revealed by the simulation calculation of density functional theory and analysis of the surface of LIBs (Figure 2c).^[^ ^53^ ^]^ Van der Waals force plays the main role in the intermolecular bonding.^[^ ^53^ ^]^ The bonding of modified PVDF is affected by two mechanisms: one involves van der Waals force that is dependent on the molecular weight of the binder, and the second is the chemical bond between PVDF and Al foil through the surface modification. The process simulation and theoretical calculation results showed that the interaction between PVDF and LiFePO_4_ particles was stronger than that of PVDF and Al foil in the LFP battery. In contrast, the interaction between PVDF and Li(Ni*_x_* Co*_y_* Mn_1_ *_‐x‐y_*)O_2_ particles in the NCM battery was weaker than that between PVDF and Al foil in the LFP battery. This is because PVDF is mainly distributed on the surface of LiFePO_4_ particles in the LFP battery, while PVDF is evenly distributed both on the Al foil and Li(Ni*_x_* Co*_y_* Mn*_1‐x‐y_*)O_2_ particles in the NCM battery. In addition, there is no chemical interaction among cathode materials, PVDF, and Al foil. The results mentioned above showed significant differences in the distribution, binding force, and action mechanism of PVDF for different cathode materials, implying that the separation efficiency for different LIBs would be considerably different even with the same separation technology.

### 2.4 Current Techniques for Quantification and Identification of PVDF

The quantity of PVDF accounts for about 1–2 wt.% of that of cathode electrodes.^[^ ^54^ ^]^ Most of the previous studies focused on the development of qualitative techniques. Because PVDF is a highly amorphous polymer, the corresponding diffraction peak in cathode electrode cannot be detected by X‐ray diffraction. However, CH_2_‐CF_2_ *_n_* characteristic peak can be detected in the C *1s* and F *1s* high‐resolution energy spectra using the X‐ray photoelectron technology, allowing the transformation of organic fluorine categories to be identified before and after separation reaction.^[^ ^55^, ^56^ ^]^ In terms of the quantitative analysis of organic fluorine, there is still a lack of precise strategy. The scanning electron microscopy‐X‐ray energy dispersive analysis technology was used for the semi‐quantitative analysis of fluorine element on the surface of the cathode electrode.^[^ ^23^, ^57^, ^58^ ^]^ Oxygen bomb combustion combined with ion chromatography can be a more accurate technology for organic halogen analysis.^[^ ^59^, ^60^, ^61^, ^62^ ^]^ The steps are as follows: first, the halogen‐containing solid material is put into a Teflon‐alloy tank with an alkaline absorption liquid at the bottom and sealed. Subsequently, the tank is filled with pure oxygen at a certain pressure from the gas bottle. The pure oxygen in the sealed tank is ignited in a calorimeter, and the content of inorganic halogen ions in the alkaline absorption solution at the bottom of the tank is measured by ion chromatography.

## 3 Different Technical Systems for the Separation of Cathode Materials and Al Foil

**Figure 3:**
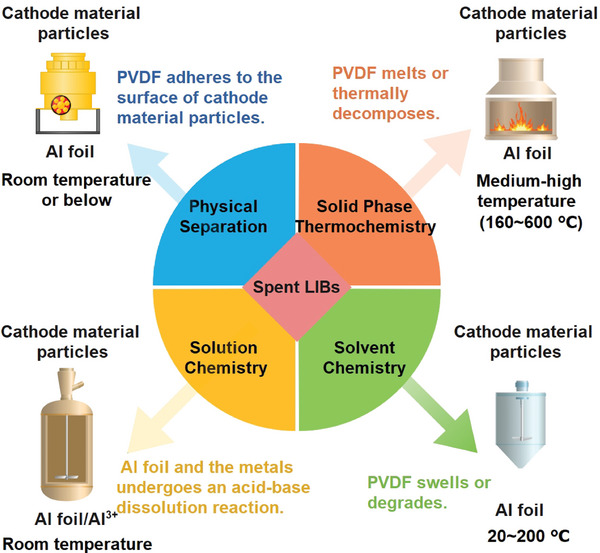
Principles of different systems for the separation of the cathode materials from Al foil.^[^ ^63^, ^64^, ^66^ ^]^

Due to differences in the design of battery recycling routes, the disposal of PVDF and the associated environmental impact are significantly different.^[^ ^63^, ^64^ ^]^ Industrial recycling process of retired LIBs involves physical crushing/screening and heat treatment.^[^ ^65^, ^66^ ^]^ Technologies in the laboratory stage include solvent dissolution, that is, NMP, for preserving the crystals in the cathode material and preparing for metal recovery or regeneration.^[^ ^67^ ^]^ Emerging technologies include pyrolysis, molten salt, electrochemistry, and supercritical fluid.^[^ ^68^ ^]^ The key technical principles involved in the separation and recycling of cathode material and Al foil can be divided into physical separation, solid‐phase thermochemistry, solution chemistry, and solvent chemistry according to different reaction media. The separation principle and general parameters of different systems are shown in **Figure** **3**.

### 3.1 Physical Separation

#### 3.1.1 Crushing and Sorting

**Figure 4:**
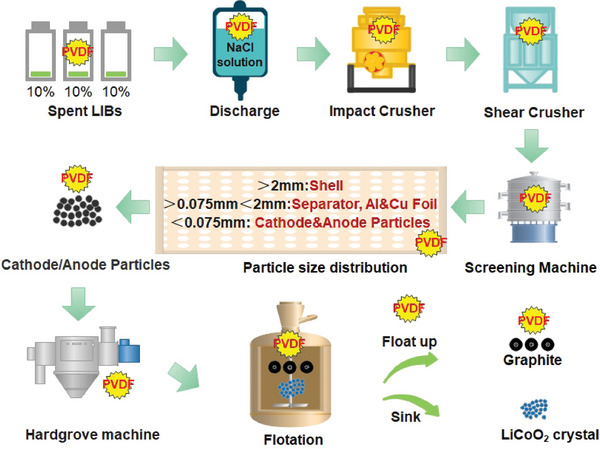
Tracing PVDF in the process of dismantling–crushing–grinding–flotation of spent LIBs.^[^ ^71^, ^75^ ^]^

Mechanical crushing and sorting refer to directly destroying the metal shell of the spent battery by external crushing force, and at the same time assisting in the separation and enrichment of electrode materials by physical methods, such as magnetic separation and gravity screening, to facilitate the subsequent pyrometallurgical or hydrometallurgical recovery of metals and non‐metallic materials. At present, this technology is widely used in engineering practice and realizes the large‐scale recycling and processing of spent LIBs.^[^ ^68^ ^]^ Separation occurs when the cathode electrode is subjected to mechanical forces in equipment such as a universal pulverizer or an impact/shear crusher.^[^ ^69^ ^]^ In LIBs, the Al foil provides flexibility and crimp elasticity. The cathode particles are evenly coated on the Al foil with PVDF as the organic binder in between to provide material stiffness. When subjected to mechanical force from a crushing action, the Al foil is sheared, inducing it to curl and allowing it to be physically separated from the cathode particles.^[^ ^70^ ^]^ As cathode materials are particulates, they can be separated from the curled Al foil by sieving or sorting through a mesh. Smelting, by contrast, directly transforms the Al foil into a recycled product. Zhang et al. discovered a selective physical crushing of each component of LCO batteries:^[^ ^71^ ^]^ under the crushing effects of both shear and impact crushers, the metal shell was enriched in the > 2 mm particle size; the separator, Cu foil, and Al foil were abundantly accumulated in the < 2 + 0.075 mm particle size; while the < 0.075 mm particle size contained the cathode/anode material particles with graphite (**Figure** **4**).

Georgi‐Maschler et al. used a ball mill and a disintegrator successively to crush the discharged spent LIBs. The milled material was classified and sorted through a vibrating screen, a drum separator, and a zigzag classifier. This process produced three types of metal‐containing material products: Fe‐Ni fraction, Al fraction, and electrode foil fraction.^[^ ^72^ ^]^ The finely mixed electrode materials of the LMO battery can be obtained by mechanical crushing and component sieving.^[^ ^73^ ^]^ Then X‐ray diffraction and other techniques can be used to characterize the material compositions and properties, providing theoretical support for the subsequent carbothermal reduction process of mixed electrode materials. In addition, ultrasonic washing has been used to assist in the post‐mechanical LIB crushing step and improve the separation efficiency of cathode material LiCoO_2_ and Al foil.^[^ ^74^ ^]^ The spent LIBs were first crushed and sieved, and the recovered materials were washed in an ultrasonic washing container with stirring. The pressure generated by the cavitation effect of the ultrasonic wave destroys the insoluble substances and disperses them in water during the washing procedure. This allows a 100% removal efficiency of cathode material from the Al foil. Moreover, the flushing effect of the agitation facilitates the separation process, reducing energy consumption and environmental pollution.

The organic film (PVDF) attached to the surface of cathode materials after the mechanical crushing and sieving form a hydrophobic organic film (Figure 4) that is difficult to remove by traditional flotation methods.^[^ ^75^ ^]^ As shown, the PVDF continues to adhere to the surface of the cathode material particles or the Al foil after mechanical separation, grinding, and flotation, suggesting that there was no decomposition or dissolution of the PVDF. Hence, the environmental impact and risks of LIB recycling will need to be considered in subsequent processing.^[^ ^76^ ^]^

#### 3.1.2 Cryogenic Grinding

In general, grinding at room temperature fails to separate the cathode material from the Al foil and tends to cause pulverization of the latter.^[^ ^77^ ^]^ According to Liu et al., the glass transition temperature (*T*_g_) of PVDF is approximately 235 K.^[^ ^78^ ^]^ The use of low‐temperature treatment can disrupt the microstructure of the cathode electrode, resulting in cracks and the layering of different sizes, which could largely enhance the detachment efficiency of the cathode material in cryogenic grinding. Consequently, the efficiency increased from 25.0 wt.% at room temperature to 87.3 wt.% after low‐temperature grinding of the cathode electrode. Metals with face‐centered cubic lattices, such as Al, reveal good mechanical properties at low temperatures.^[^ ^79^ ^]^ In addition, the dramatic difference in particle size permits the crushed products to be separated by a simple sieving operation.

The above results demonstrate that despite the general feasibility of the physical separation of cathode material particles and Al foil by a mechanical‐physical method, the following processing issues need to be further addressed.^[^ ^33^, ^80^, ^81^ ^]^ 1) The strong bonding effect of organic binder PVDF results in inefficient release of the cathode material by mechanical‐physical methods. After physical crushing and sieving, a small number of the cathode material particles continued to adhere on the surface of the Al foil, causing quality loss and additional challenges in Al foil smelting. Therefore, to improve the efficiency of subsequent hydrometallurgy, the calcination of cathode material powder to remove PVDF is essential. 2) The superior chemical stability of PVDF on the surface of the cathode material particles after separation may induce negative impact on the environment and ecology, which should be considered in the subsequent metal recycling. 3) The cross‐contamination of crushed Al chips with cathode material particles needs to be addressed, particularly when prolonged crushing or excessive milling speed are used. The high content of Al adversely affects the extraction and separation of metals in the follow‐up hydrometallurgy. Therefore, controlling the crushing time is crucial for achieving efficient hydrometallurgical extraction of the target metallic fractions.

### 3.2 Solid Phase Thermochemistry

**Figure 5:**
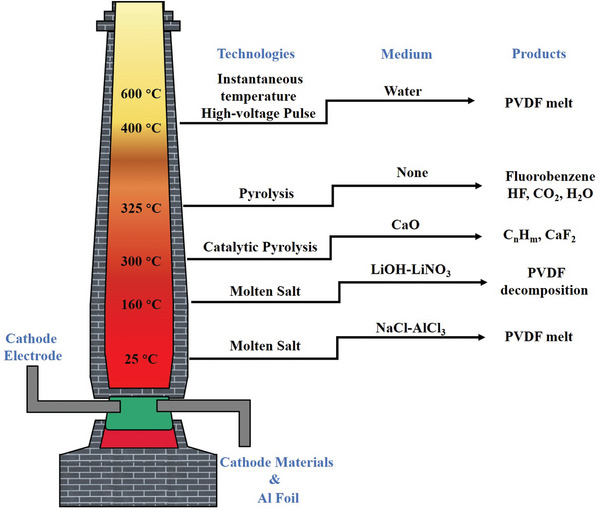
Degradation products and occurrence forms of PVDF in solid phase thermochemical media at different temperatures.^[^ ^23^, ^82^, ^84^, ^90^, ^91^ ^]^

Solid phase thermochemistry refers to melting or degrading PVDF in the cathode electrode by high‐temperature thermochemical reaction. Thermal treatment in an oxygen atmosphere is called calcination or incineration, while thermal treatment in an oxygen‐free or inert gas atmosphere is viewed as a pyrolysis process.^[^ ^82^, ^83^ ^]^ Direct calcination of PVDF can easily cause the emission of pollutants, while a fully enclosed environment for the pyrolysis process can better control and treat the polluting gases.^[^ ^84^, ^85^, ^86^ ^]^ Specifically, this method can directly pyrolyze and convert PVDF into pyrolytic oil, gas, and hydrogen fluoride (HF).^[^ ^87^, ^88^ ^]^ Meanwhile, alkaline catalyst can accelerate catalytic degradation of PVDF and enable in‐situ capture of inorganic fluoride.^[^ ^23^ ^]^ To eliminate the generation of dioxins, ammonia injection was considered as an effective strategy for high‐temperature dehalogenation.^[^ ^89^ ^]^ Apart from direct and catalytic pyrolysis, new technological approaches, such as molten salt‐mediated treatment and pulsed high‐voltage discharge, can achieve the melting or degradation of PVDF in solid thermal media, detaching the cathode material from the Al foil and preventing the release of fluoride (**Figure** **5**).^[^ ^90^, ^91^ ^]^

#### 3.2.1 Calcination/Pyrolysis

Pyrolysis enables the decomposition of PVDF by placing the cathode electrode into a high‐temperature incinerator or rotary kiln under inert gas protection, achieving separation between cathode materials and Al foil.^[^ ^83^ ^]^ **Table** **1** summarizes the current heating processes for cathode materials and Al foil separation. It should be noted that the melting point and thermal decomposition temperature of PVDF are approximately 160 and 300 °C, respectively; hence high‐temperature treatment is a convenient and efficient method for the separation of LIB components.^[^ ^92^, ^93^ ^]^

**Table 1:** Thermochemical processes for separation of cathode materials and Al foil

Process	Samples	Atmosphere	Reaction Conditions	Separation Percentage	Products	References
Calcination	NCM	Air	450°C, 2 h	100%	Li(Ni_1/3_Co_1/3_Mn_1/3_)O_2_	[94]
	NCM	Air	500°C, 1.5 h	97.1%	LiNi_0.33_Mn_0.33_Co_0.33_O_2_	[82]
	NCM	Air	600°C, 5 h	100%	Li(Ni_1/3_Co_1/3_Mn_1/3_)O_2_/C	[95]
	LFP	Air	600°C, 1 h	100%	Li_3_Fe_2_(PO_4_)_3_/Fe_2_O_3_	[96]
	Cathode mixture	Air	300°C, 1 h, then 550°C, 0.5 h	100%	LiCoO_2_/Co_3_O_4_/Li_4_Mn_5_O_12_/Li_0.9_Ni_0.5_Co_0.5_O_2‐x_	[97]
	Cathode mixture	Air	550°C, 1.5 h	99.01%	LiCoO_2_/LiNiO_2_/LiMn_2_O_4_	[98]
Pyrolysis	LFP	N_2_	500°C, 0.5 h	96.86%	LiFePO_4_/C	[99]
	NCM	N_2_	600°C, 15 min	100%	Li(Ni_1/3_Co_1/3_Mn_1/3_)O_2_	[100]
	LFP	N_2_	550°C, 2 h	99.34%	LiFePO_4_/C/Al	[101]
	LCO	N_2_	500°C, 15 min	98.23%	LiCoO_2_/C	[102, 103]
	NCM	N_2_	450°C, 0.5 h	100%	Li(Ni_1/3_Co_1/3_Mn_1/3_)O_2_/Al	[104]
	Cathode mixture	N_2_	550°C, 1.5 h	97.90%	LiCoO_2_/LiNiO_2_/LiMn_2_O_4_	[98]
Vacuum Pyrolysis	LCO	<1.0 kPa	600°C, 0.5 h	100%	LiCoO_2_, CoO, and Al foil	[105, 106]
	LCO	Vacuum	600°C, 3 h	100%	Cathode powder, and Al, Cu and Ni, etc.	[107]
	LCO	Vacuum	500°C, 15 s	99.78%	LiCoO_2_/C	[108]
	LCO	N_2_, Vacuum	500°C, 0.5 h	100%	LiCoO_2_/C	[109]
	NCM	1000Pa	600°C, 0.5 h	98.04%	LiNi_x_Co_y_Mn_1‐x‐y_O_2_/NiO_2_	[110]
	LCO	Vacuum	673 to 723 K, 1 h	100%	LiCoO_2_, CoO, and Al foil	[111]
	LFP	1000 Pa	600°C,0.5 h	100%	LiFePO_4_/C	[112]
	Cathode mixture	N_2_, Vacuum	650°C, 1.5 h	97.90%	Li_2_CO_3_/Mn_3_O_4_/Co_3_O_4_/NiO	[98]

Research results showed that PVDF in the cathode electrode initially decomposed at a heating temperature of 380 to 400 °C. At 600 to 700 °C, the conductive additive reacted with oxygen by combustion, at which the Al foil melted (melting point of 660 °C).^[^ ^82^ ^]^ Another study showed that when fragments of a waste LCO battery were placed in a vacuum heating chamber at 600 °C, the PVDF achieved complete pyrolysis after heating for 30 min, but the conductive additive did not combust.^[^ ^105^ ^]^ As the heating temperature failed to meet the standard of decomposition of cathode materials, the LiCoO_2_ crystal and the conductive additive could not realize complete shedding from the Al foil. Christian et al. examined the pyrolysis rate of PVDF at different temperatures and found that PVDF began to decompose at 350 °C, reached a weight loss of 98.9 wt.% at 550 °C, and totally decomposed at 580 °C. Thus, the optimal pyrolysis interval for PVDF is 500–580 °C.^[^ ^82^ ^]^ Furthermore, the experimental results confirmed that the ideal pyrolysis temperature for the organic binder in the electrode material was 500 °C, and the primary pyrolysis products were fluorobenzene, hydrocarbon, and ester.^[^ ^102^, ^113^ ^]^ Upon pyrolysis, the release efficiencies of the cathode material rose from 82.9 to 99.8 wt.%. Other research has also confirmed that the electrolyte was the primary source of the fluorine‐containing gases, and that HF was released during the pyrolysis of the spent LIBs.^[^ ^87^, ^112^, ^114^ ^]^ The PVDF binder decomposed into H_2_O, CO_2_, and fluorine‐containing gases, with the largest peak detected at 522 °C.

Moreover, Liu et al. investigated high‐temperature separation of the cathode electrode in a retired LFP battery and carried out a kinetic study of the pyrolysis process, employing the iso‐conversional method.^[^ ^115^ ^]^ The results revealed that the average pyrolysis activation energy of the cathode electrode was 85.36 kJ mol^−1^, which depended on the temperature zone. Under nitrogen protection, 50 wt.% of the PVDF decomposed and was converted to pyrolysis gas and oil phase at about 475 °C, whose main components were HF and light hydrocarbons, and the HF gases could be absorbed by alkaline solution. Furthermore, graphite and LiFePO_4_ crystals retained their material and structural integrity under this condition. Huang et al. proposed that the co‐pyrolysis of electrolytes, PVDF, and active materials exhibited significant adverse synergistic effects.^[^ ^32^ ^]^ In the co‐pyrolysis process, active materials adsorbed the electrolyte residues on the surface and into the pores (20–200 °C), while PVDF formed a liquid film that covered the local surface of the active materials (400–500 °C). These interactions prevented the efficient removal of organics, leaving fluorine‐containing contaminants in the active materials. Enhancing the pyrolysis temperature can facilitate the removal of organics.

These recent studies have shown that a degradation temperature ca. 500 °C is relatively reliable for PVDF degradation. In this process, the production of fluorine‐containing organics such as fluorobenzene and hydrocarbons was detected. These fluorinated organic pollutants are a potential source of environmental pollution and a risk factor in applying pyrolysis technology. Differences in thermochemical strategies may affect the compositions and pathways of the degradation products of PVDF. However, relevant research is still insufficient.

To eliminate the negative environmental impact of PVDF in the thermochemical reaction, calcium oxide (CaO) was used as an alkaline defluorination medium. The results concluded that PVDF in the cathode electrode could undergo catalytic pyrolysis and de‐fluoridation at a temperature of 300 °C.^[^ ^23^ ^]^ The pyrolysis oil‐phase products were hydrocarbons, and the pyrolytic gas was hydrogen. The carbon dioxide attained in situ adsorption and immobilization by CaO. The released HF gas was converted to calcium fluoride by CaO. The separation efficiency of the cathode material from the Al foil under optimal conditions exceeded 97.1 wt.%. Consequently, the employment of CaO not only reduced the energy consumption but also averted the release of HF, presenting both economic and environmental benefits.

#### 3.2.2 Molten Salt‐Mediated Treatment

Molten salt is a liquid reaction medium formed by heating an ordinary solid inorganic salt above its melting point (e.g., NaNO_3_ melts at 308 °C, NaCl melts at 801 °C), followed by a series of physical and chemical reactions including heat storage, oxidation, and electrolysis, utilizing its properties as a thermal medium.^[^ ^116^ ^]^ As an anhydrous high‐temperature flux, the main feature of molten salt is its dissociation into ions when melted, and the positive and negative ions are bound together by Coulombic forces, making it available as a thermal reaction medium at high temperatures.^[^ ^117^, ^118^ ^]^ Considering the advantages of molten salt, such as a wide range of operating temperature, high thermal conductivity, low working pressure, and low material cost, it has been broadly studied in the field of solid waste treatment.^[^ ^119^, ^120^ ^]^

A low‐temperature molten salt, an AlCl_3_/NaCl molten salt system, was first proposed for separating spent cathode materials and Al foil.^[^ ^90^ ^]^ The findings showed that the AlCl_3_/NaCl molten salt system allowed for efficient and rapid melting of PVDF, compared to KNO_3_/NaNO_3_ or KOH/NaOH molten salt. The former held a melting point of 153 °C, and its phase transition from solid to liquid absorbed a large amount of heat, resulting in the melting of PVDF. When the temperature fell to room temperature, the heat stored in the AlCl_3_/NaCl molten salt was released, and the medium reverted to the solid state. The phase transition repeatedly circulated to a heat storage and release cycle, which was repeated as necessary, to separate the cathode materials and Al foil. The optimum separation conditions were temperature of 160 °C, holding heating time of 20 min, and mass ratio of molten salt/cathode electrode of 10:1. In this process, the highest percentage of detachment of Al foil that could be achieved was over 99.8 wt.%. After molten salt treatment, the organic fluorine species adhering to the surface of the cathode materials remained in the form of PVDF. Therefore, the high‐temperature melting of PVDF is considered to be the reason for the separation of Al foil and cathode materials. Ji et al. studied the separation efficiency of binary eutectic systems of three common inorganic lithium compounds — LiCl, LiOH, and LiNO_3_ — on cathode materials and Al foil.^[^ ^121^ ^]^ Among the three binary mixtures, LiNO_3_‐LiOH exhibited the best detachment performance. The cathode materials (NCM111 and LiMO) achieved a detachment efficiency of 98.3 wt.% with Al foil at a LiNO_3_‐LiOH molar ratio of 2:3, temperature of 260 °C, duration of 30 min, and salt‐to‐cathode mass ratio of 10:1. As for the other cathode electrodes — LCO and LFP — the detachment efficiencies of LiNO_3_‐LiOH were 95.7 and 87.8 wt.%, respectively.

Current studies indicate that since the melting point of PVDF is 160 °C, the range of molten salt utilization is between 160 and 300 °C, which is much lower than the complete thermal decomposition temperature of PVDF (400–500 °C). Molten salt separation presents significant environmental benefits such as low energy consumption, low reagent cost, free discharge of wastewater, and excellent recyclability. To date, reports on molten salt exfoliation of cathode electrode sheets are limited. Depending on the molten salt system, the separation mechanisms of Al foil and cathode materials differ considerably. Overall, at a low temperature (160 °C), PVDF melting is believed to be the cause of the exfoliation of the cathode materials, while at a relatively high temperature (300 °C), if an alkaline medium exists, the PVDF is likely to be degraded through the nucleophilic substitution of alkaline groups, resulting in the separation of cathode materials and Al foil. Considering its environmentally friendly properties and relatively low operating temperature, the molten salt process is deemed as a new detaching technology for battery cathode electrode with promising industrial application.

#### 3.2.3 High‐Voltage Pulse

High‐voltage pulse takes advantage of pulsed power technology to emit massive amounts of energy over a brief period, dissociating metallic conductive materials from non‐metallic non‐conductive materials in solid materials. This technology has successfully separated metallic and non‐metallic composites in urban minerals.^[^ ^122^ ^]^ Motivated by the conductivity of Al foil and the non‐conductivity of cathode material particles/graphite, a novel electrical method for separating cathode materials and Al foil, based on pulsed high‐voltage discharge, was developed.^[^ ^91^ ^]^ First, the cathode material was cut into a 30 mm × 80 mm test sheet and discharged with a high‐voltage electric pulse, using water as a protective medium. At a voltage of 25 kV, 93.9 wt.% of the cathode material particles were separated from the Al foil. The Al foil remained intact after separation, and the cathode material particles could be recovered after sieving through a 2.36 mm mesh. As per the mechanistic analysis, the PVDF binder in the cathode material particle layer melted and lost adhesion by Joule heating of the Al foil under the action of electric current, while the recovered cathode particles maintained their chemical composition and shape after separation. In addition, life cycle assessment revealed the potential to reduce greenhouse gas emissions and resource consumption by recovering cathode material particles and Al foil from cathode electrodes through pulsed discharge when the disposal scale was sufficiently large.^[^ ^123^ ^]^ Overall, the technology is simple and effective, providing rapid separation of the cathode material from the Al foil, thereby obviating the risk of environmental contaminant releases.

Thermal treatment has application advantages including the simplicity of the process, ease of operation, and extensive scale‐up options. By adjusting the temperature interval and selecting the reaction medium, the method allows for adjustable melting or decomposition of PVDF, making it the most popular choice for separating cathode materials and Al foil in recovering retired power LIBs. In industrial application, high‐temperature pyrolysis is the most mature and straightforward method for batch treatment of the PVDF remaining on the surface of cathode material particles after physical sorting. However, the pyrolysis process can release HF gases, leading to noticeable challenges, including atmospheric pollution, volatilization of active Li, and equipment corrosion. Although alkali absorption tackles the exhaust pollution, the intense corrosiveness of inorganic fluoride tends to shorten the working life inside high‐temperature treatment equipment and increases recovery costs. Moreover, dioxin, a derivative of organic fluoride, would incur latent environmental hazards.

From the perspective of battery classification, for a retired LFP battery, the high energy consumption of heat treatment often squeezes the profit margin of the Li extraction while increasing carbon emissions, resulting in an unprofitable LFP battery recycling situation. The high carbon emissions also counteract the original intention of promoting new EVs. Therefore, high‐temperature treatment is not a sustainable route for the recycling of spent LFP batteries. The concomitant separation of cathode materials and Al foil via the hydrometallurgical route appears to be more cost‐effective and low‐carbon. For LCO and NCM batteries, corrosive‐resistant materials should be substantially developed for practical heat recovery, along with tail gas‐absorption devices to strictly control the amount and concentration of fluoride releases and to closely monitor the dioxins to prevent secondary pollution.

### 3.3 Solution Chemistry

**Figure 6:**
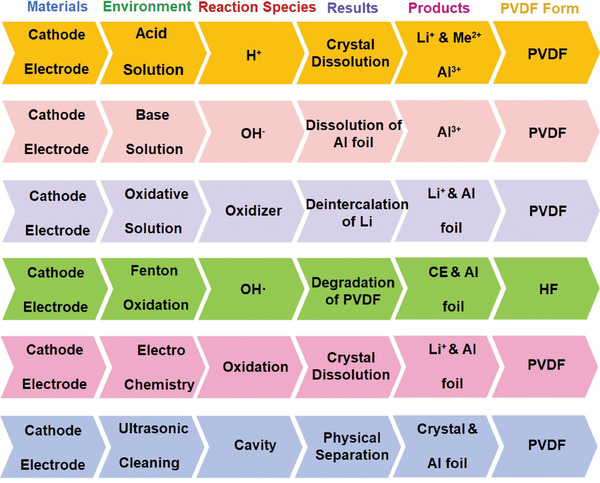
Mechanisms and reactive species of separation of cathode material and Al foil with different solution systems.^[^ ^124^, ^125^ ^]^

Regulating the solution chemistry conditions in an aqueous environment can efficiently separate cathode materials from Al foil. There are various ways to meet this objective, including acid/base/oxidative dissolution, Fenton oxidation, electrolysis, and ultrasonic cleaning (**Figure** **6**).^[^ ^124^, ^125^ ^]^ These processes proceed in aqueous solutions, but the separation mechanisms vary. Acid dissolution enables separation by moderating the acidity of the solution to dissolve the cathode material particles and leach Li to weaken the chemical bonding of the PVDF. Alkali dissolution realizes the detachment of cathode material particles by selective dissolution of Al foil. Fenton oxidation involves depolymerizing PVDF molecules by free radical reaction, accomplishing material separation. Ultrasonic cleaning utilizes material vibration triggered by the cavitation effect to weaken the adhesion between cathode materials and Al foil.

#### 3.3.1 Acid/Base/Oxidative Separation

Acid/base/oxidative dissolution implemented in an aqueous solution does not affect organic polymer PVDF but separates the cathode material and the Al foil by different mechanisms.^[^ ^126^ ^]^ The base system consists of selective dissolution of the Al foil, because the cathode material particles (LCO and NCM) generally do not react with the alkaline solution, while the current collector Al foil does.^[^ ^127^ ^]^ NaOH (5 wt.%) is used as the dissolving solution for the Al foil of the cathode electrode.^[^ ^128^ ^]^ Almost all the Al foil dissolves in the alkaline solution at room temperature, while cathode material powder is obtained after filtration.

The acid dissolution method aims to directly dissolve all the valuable metals in the cathode material, such as Li, Ni, Co, and Mn, in an acidic solution, followed by solvent extraction or fractional precipitation to separate and recover the different metals.^[^ ^125^, ^129^ ^]^ During this process, high acid concentration will cause the dissolution of the Al foil. Given the above process defects, organic trifluoroacetic acid (TFA) was adopted as a leaching reagent to separate cathode materials and Al foil in the waste LIBs.^[^ ^130^ ^]^ Under the optimal conditions of 15 vol.% of TFA solution, L/S ratio of 8.0 mL g^−1^, temperature of 40 °C, and time of 180 min, the Li could be selectively leached, weakening the bonding of the PVDF to the crystal. The Al foil prevented the corrosion and dissolution of the TFA. As a result, complete separation of cathode material and Al foil was achieved. In this recycling process, PVDF is considered to still exist on the surface of the cathode material after hydrometallurgical leaching. These organic binders can be removed by calcination at the temperature of 700 °C.

As outlined in Section 2.4, the mechanisms of action of PVDF differ markedly among the different cathode electrode types. Compared to NCM and LCO crystals, PVDF interacts more feebly with Al foil in the LFP cathode electrode.^[^ ^131^ ^]^ Accordingly, automatic separation of the C/FePO_4_ residue from the Al foil has been developed using a feasible design process after selective Li extraction.^[^ ^132^ ^]^ Yang et al. suggested a route for selective leaching of Li from retired LFP batteries by regulating the proton acid concentration and separating out the Al foil.^[^ ^133^ ^]^ The Al foil stayed undissolved into the liquid phase with reasonable control of the proton acid concentration in the acetic acid solution. At the same time, the Li in the LiFePO_4_ could be selectively extracted, owing to the mosaic effect of the Li in the olivine structure of the FePO_4_. After selective leaching of Li, the C/FePO_4_ residue was separated from the Al foil. Zhang et al. confirmed that after the Li in the retired LFP battery was extracted by oxidation, the bond between the C/FePO_4_ residue and the Al foil decreased, thus enabling the residue separation from the intact Al foil.^[^ ^134^, ^135^, ^136^ ^]^ In the hydrometallurgy process, PVDF was located on the surface of graphite residue and FePO_4_ skeleton, and only an amount of inorganic fluorides entered the leaching solution in the form of lithium fluoride.

The separation of cathode materials and Al foil by acid/base/oxidative dissolution consumes considerable amounts of concentrated alkali or acid and generates a massive quantity of wastewater, adding to the recovery cost. In practical hydrometallurgy, dissolving Al foil into the liquid phase before precipitation separation is not a preferable approach. The aqueous solution separation method requires high corrosion protection for the equipment and causes the dissolution of highly reactive Li, rendering it impractical for large‐scale batch processing. The development of organic acids has relieved the environmental pressure and burden, yet these acids are expensive. Furthermore, mixed types of metals introduced into the liquid phase will require tedious subsequent metal separation steps.

#### 3.3.2 Fenton Oxidation

Fenton oxidation adopts hydrogen peroxide (H_2_O_2_) and ferrous (Fe^2+^) reagents to generate hydroxide radicals in a chemical reaction to obtain the oxidative degradation of organic pollutants.^[^ ^137^, ^138^ ^]^ This method has been broadly applied to the decomposition of organic matter in the atmosphere and soil.^[^ ^139^, ^140^ ^]^ Following physical and mechanical crushing, PVDF remains attached to the surface of cathode material particles and graphite particles and forms a hydrophobic organic membrane. Traditional methods struggle to effectively separate cathode materials and graphite, creating an obstacle for subsequent metal recovery.^[^ ^35^ ^]^ As PVDF is essentially a linear polymer with simple chemical composition and structure, Fenton oxidation could be applied to degrade PVDF on the surface of LCO cathode materials and graphite into water‐soluble inorganic fluorine, phosphate, carbonate, and other small‐molecule organic compounds, which solved the surface residue problem of PVDF in an efficient and eco‐friendly way.^[^ ^141^ ^]^

However, this method is applicable for dealing with a small volume, and costs more for reagents. Introducing impurities such as iron into the system potentially creates unfavorable interfering factors for the subsequent hydrometallurgical extraction of the critical metals.^[^ ^142^ ^]^ The above considerations account for the limited extent of practical application of Fenton oxidation in the decomposition and recovery of PVDF. For spent LFP battery recycling, Fenton oxidation stripping may be more suitable. Wu et al. used the Fe^2+^ in waste LiFePO_4_ crystal to induce an in‐situ Fenton reaction, developed a green and economical H_2_O_2_ system, and realized the complete recycling of waste LFP batteries.^[^ ^143^ ^]^ The optimal conditions were H_2_O_2_ concentration of 0.2%, rotation speed of 400 rpm, reaction time of 4 min, and solid–liquid ratio of 14.2 g L^−1^. The loss of Al foil was less than 0.4 wt.%. The success of this application could be attributed to the weaker binding force of PVDF to Al foil in the LFP cathode electrode (as described in Section 2.3). Mechanistic analysis demonstrates that in a Fenton system, hydroxyl radicals can rapidly oxidize part of the PVDF, thereby reducing the binding energy between PVDF and Al foil and weakening the molecular binding force between Al foil and cathode materials. According to He et al., the degradation products of PVDF after Fenton or electrolytic oxidation were likely to be small molecules.^[^ ^141^ ^]^ Compared to traditional methods, Fenton oxidation system has the advantages of zero acid, zero base, or zero organic solvent usage and mild stripping conditions. However, the feasibility of this method for separating NCM and LCO batteries is questionable.

#### 3.3.3 Electrochemistry

Electrochemistry describes the process of separating cathode materials from Al foil through a chemical reaction in the presence of an applied current. Similar to Fenton oxidation, electrochemistry may be more applicable to the peeling of cathode materials from Al foil of LFP batteries due to the relatively weak binding force between PVDF and Al foil. In an electrochemical oxidation technique designed by Wu et al., only 3 g L^−1^ sodium sulfate was used as the electrolyte, the LFP cathode material could be completely oxidized and stripped within 15 min under the electrolysis voltage of 10 V.^[^ ^143^ ^]^ Morphology and crystal structure analysis showed that the LFP cathode material was completely exfoliated, the Al foil was clean, and there was no corrosion.

Considering the complexity of the cathode electrode, electrolytic separation is not a technology targeted at separation; instead, it serves to accompany the recovery of metal compounds. As for advantages, it enables the separation to obtain pure Al foil and induces electrolytic conversion of partial cathode material to ionic form for the follow‐up precipitation step. Nevertheless, the disadvantage lies in the thicker coating of cathode material particles on the Al foil surface, which may cause low electrolysis efficiency. The heavy electricity consumption also incurs high cost.

#### 3.3.4 Ultrasonic Cleaning

Ultrasonic propagation can vibrate the media molecules and lead to a cavitation effect through bubbles, weakening the bond between the cathode material and the Al foil and thus achieving separation. However, direct sonication of the cathode electrode proves to be exceedingly difficult for achieving detachment for LFP batteries. Auxiliary solution media, such as LiOH solution,^[^ ^144^ ^]^ inorganic acid,^[^ ^145^ ^]^ organic acid,^[^ ^146^ ^]^ Fenton reagent,^[^ ^147^, ^148^ ^]^ and organic solvent,^[^ ^149^ ^]^ are essential to attenuate the adhesion of the organic binder. The detachment efficiency can be improved dramatically with acidic solutions (sulphuric acid and oxalic acid) as the media. Approximately 99, 100, and 46 wt.% of the coating cathode material could be separated in sulphuric acid, oxalic acid, and pure water media, respectively, yielding Al foil with corresponding purities of 98, 99, and 15 wt.%. A plausible explanation for this accomplishment could be the strong oxidation of HO· radicals formed in the acidic ultrasonic solution leading to the quick degradation of the adhesive. In summary, this approach enhances the detachment efficiency and product purity of the cathode materials and Al foil, presenting an alternative solution for the recovery of retired LIBs.

Hydrometallurgy remains the dominant technology for metal recovery from the cathode materials, followed by solution chemistry, which is a highly effective method for separating cathode material from Al foil and degrading PVDF. The tunability is the advantage of applying a solution chemical system for separating cathode materials and Al foil. That is, by choosing a suitable acid, alkali, or oxidation system, dissolution of the Al foil, leaching of metals, and/or selective recovery of Li can be flexibly achieved. However, solution chemistry is not currently a prevailing separation strategy for the following reasons: 1) possible dissolution of Al foil during the separation process prolongs the metal recovery and poses impurity separation challenges; 2) generation of acid/alkali wastewater boosts reagent consumption and metal recovery costs; and 3) crystalline compounds may undergo corrosion in solution chemistry, entailing the loss of valuable metals.

### 3.4 Solvent Chemistry

Due to the release of organic fluorides and the possible formation of dioxins, thermochemical defluorination is a less‐than‐perfect response to the challenge of separating cathode materials from Al foil.^[^ ^150^ ^]^ In contrast, solvent chemistry is likely to be a greener and more environmentally friendly measure. Compared with direct high‐temperature thermal treatment, solvent chemistry decomposes PVDF under milder reaction conditions, i.e., 20–200 °C, and consumes relatively less energy. Secondly, solvent chemistry can address the problem of the release of organic fluoride.^[^ ^151^ ^]^ The decomposition of PVDF encompasses the recovery or removal of organic solvents, ionic liquids (ILs), deep eutectic solvents (DESs), or bio‐based solvents, and includes supercritical fluid extraction (**Table** **2**).

**Table 2:** Solvent processes for separation of cathode materials and Al foil

Solvents		Cathode materials	Reaction Conditions	Separation percentage	Products	References
Organic solvents	*N*‐methyl‐2‐pyrrolidone	LCO	Room temperature, ultrasonic treatment, 3 min	99%	Al foil, C, LiCoO_2_	[152]
	*N*, *N*‐dimethylformamide	LCO	60°C, 2 h	100%	Al foil, C, PVDF, LiCoO_2_	[153]
	Dimethylacetamide	NCM	84°C, 5 min	97%	Al foil, C, PVDF, LiNi_1/3_Co_1/3_Mn_1/3_O_2_	[154]
	Ethylene glycol	NCM	S/L mass ratio = 1:10, 160°C, 6 s	100%	Al foil, C, PVDF, LiNi_0.5_Mn_0.3_Co_0.2_O_2_	[155]
	Triethyl phosphate	NCM	S/L mass ratio = 1:10, 100°C, 1 h	100%	Al foil, C, PVDF, LiNi_0.6_Mn_0.2_Co_0.2_O_2_	[156]
	Cyrene	NCM	S/L ratio = 500 g L^−1^, 100°C, 1 h	100%	Al foil, C, PVDF, LiNi_0.6_Mn_0.2_Co _0.2_O_2_	[157]
	Dimethyl isosorbide	Cathode electrode mixture	S/L ratio = 1:40 g mL^−1^, 150°C, 5 h	100%	Al foil, cathode materials	[158]
	Glycerin	NCM	200°C, 350 r min^−1^, 15 min	95%	Al foil, C, PVDF, LiNi_0.5_Co_0.2_Mn_0.3_O_2_	[159]
IL	1‐Butyl‐3‐methyl‐imidazolium‐tetrafluoroborate	LCO	300 r min^−1^, 180°C, 25 min	99%	Al foil, C, PVDF, LiCoO_2_	[160]
DES	Choline chloride–glycerol DES	NCM	190°C, 15 min	99.86%	Al foil, C, PVDF, Li(NiCoMn)_1/3_O_2_	[57]
	Potassium carbonate–ethylene glycol DES	LFP	S/L mass ratio = 1:20‐25, 100°C, 20 min	99.3%	Al foil, C, PVDF, LiFePO_4_	[161]
Biological solvents	Citrus juice	LCO	S/L ratio = 50 g L^−1^, 90°C, 20 min	100%	C, Al, LiCoO_2_, Co_3_O_4_	[162]
	Waste oil‐based fatty acid methyl esters	LFP	190°C, 20 min	99.1%	Al foil, C, PVDF, LiFePO_4_	[58]
Supercritical fluid	Supercritical CO_2_	LCO	10 MPa, 38 °C, 15 min	98.86%	Al foil, C, LiCoO_2_	[163]
	Supercritical CO_2_ & dimethyl sulfoxide	NCM	8 MPa, 70 °C, 13 min	98.5%	Al foil, C, Li(NiCoMn)_1/3_O_2_	[164]

#### 3.4.1 Organic Solvents

In LIB production, the intensive polar solvent NMP is generally preferred for dissolving PVDF, followed by homogeneous mixing of the dissolved PVDF solution with cathode materials to coat the binder and the cathode material in the Al foil.^[^ ^67^, ^165^, ^166^, ^167^ ^]^ Hence, according to the principle of “similar miscibility”, organic solvents with strong polarity can effectively dissolve or swell PVDF for cathode material separation and Al foil recovery. The solubility of PVDF in three selected organic solvents (*N*‐dimethylformamide (DMF), *N*‐dimethylacetamide (DMAC), and NMP) was found to be positively correlated with temperature.^[^ ^153^ ^]^ When the temperature reached 70 °C, the solubilities of PVDF in DMAC, NMP, and DMF were 214, 216, and 176 g L^−1^, respectively. The cathode material and Al foil could then be completely separated and recovered.

The dissolution effects of four different organic solvents (DMAC, NMP, DMF, and dimethyl sulfoxide (DMSO)) on PVDF in the cathode electrode of a retired NCM battery were compared, and the results showed that DMAC, which is low‐toxic, environmentally friendly and cost‐effective, could achieve the optimal separation.^[^ ^154^ ^]^ After processing at 60 °C for 85 min, clean and bright Al foil was obtained and applied directly in smelting. The dissolution of PVDF in NMP cleaning solution, followed by ultrasonic cavitation, efficiently separated the cathode material from the Al foil, with a simultaneously decreasing agglomeration of the cathode material particles, which facilitated the subsequent leaching process.^[^ ^168^ ^]^

Despite the relatively effective adoption of organic solvents to dissolve PVDF and their frequent application in the laboratory stage, the use of organic solvents—particularly at a large scale—needs to consider hazard risks because of their volatility and high toxicity, which can pose substantial hazards to occupational health and the environment. Accordingly, to weaken the potential toxicity of solvent chemistry, He et al. developed an exfoliating extract whereby the cathode material could be detached from the Al foil by weakening the mechanical interlocking force and the Coulombic force between them.^[^ ^169^ ^]^ The recovery rates of electrolyte, Al foil, Cu foil, and cathode materials were 95.6, 99.0, 100, and 100 wt.%, respectively. As a result, the exfoliating extract effectively prevented impurities from penetrating cathode materials. However, the ingredients of the exfoliating liquid have so far not been disclosed due to patent protection.

Some new and low‐toxic organic solvent systems, such as ethylene glycol,^[^ ^155^ ^]^ triethyl phosphate,^[^ ^156^ ^]^ cyrene,^[^ ^157^ ^]^ and dimethyl isosorbide,^[^ ^158^ ^]^ have been developed for the separation of cathode materials and Al foil. In the reports published so far, there is still no consensus on the separation mechanisms of cathode materials and Al foil in these organic solvent systems. Overall, the dissolution mechanism of PVDF in dimethyl isosorbide proposed by Buken et al. can be referred to.^[^ ^158^ ^]^ PVDF adheres to the current collector Al foil through hydrogen bonds. The rapid dissolution of PVDF in organic solvents can be attributed to the following steps: i) dimethyl isosorbide first penetrates the PVDF crystalline region to reduce the PVDF polymer chain interactions, ii) dimethyl isosorbide interacts with hydrogen and fluorine atoms in PVDF and establishes hydrogen bonds to enhance solvent‐polymer interactions; iii) dimethyl isosorbide actively participates in the formation of hydrogen bonds in the thin oxide layer of the alumina coating of the current collector, thereby weakening the PVDF adhesion in the Al foil and promoting the swelling and partial dissolution of PVDF, thus effective separation of cathode materials. However, the potential environmental toxicity of the organic solvents is an unavoidable and hidden danger in practical application. The expensive and toxic organic solvents also increase the recovery costs. Therefore, the separation of cathode materials by organic solvents remains at the laboratory stage and fits small‐scale processing or material regeneration rather than large‐scale industrial application.

#### 3.4.2 Ionic Liquids

Inorganic or organic anions and cations constitute ILs, which are solvents containing ions with properties of high electrical conductivity, low melting point, superior thermal stability, and outstanding solubility.^[^ ^170^ ^]^ ILs in various structural forms composed of different anions and cations can dissolve many organic and inorganic compounds, polymeric materials, etc. The imidazolium IL was first used to address the challenge of separating the cathode material and Al foil in the retired LIBs.^[^ ^160^ ^]^ The detachment rate of the cathode material was low as the temperature fell below 165 °C (speed of 300 rpm and duration of 20 min). Given that 165 °C is lower than the melting temperature of PVDF, the small amount of detachment likely came from damage by the mechanical forces of the mixer. Thus, the most favorable separation temperature was determined to be 180 °C, and the reaction of the cathode electrode in the imidazolium IL was considered to be the dissolution of PVDF.

There have been numerous reports on applying ILs to e‐waste.^[^ ^171^ ^]^ Apart from tackling the separation of waste LIB cathode materials and Al foil, researchers studying the recycling of waste printed circuit boards,^[^ ^172^, ^173^, ^174^ ^]^ fluorescent powder,^[^ ^175^, ^176^, ^177^ ^]^ and so on have observed the success of exploiting ILs. However, the high fluid viscosity makes ILs prone to adhesion on the surface of cathode materials, resulting in mass loss.^[^ ^178^ ^]^ Due to the high synthesis cost of ILs, the recovery expense is likely to increase. Moreover, the potential adverse impact of ILs as reaction media on the ecological environment remains unclear, representing a practical obstacle for ILs to be promoted in the industrialization of spent LIB recycling.

#### 3.4.3 Deep Eutectic Solvents

The high cost, sophisticated synthesis steps, and potential biological toxicity may limit broad application of ILs. In this regard, there is a direct need to develop cost‐effective, efficient, and minimally toxic green solvent systems to degrade PVDF in the retired LIBs. Compared with ILs, inexpensive and broadly available DESs are preferred. DESs are two‐ or three‐component eutectic mixtures consisting of a certain stoichiometric ratio of hydrogen bond acceptor and hydrogen bond donor, whose freezing point falls dramatically below the melting point of the pure substances of each component.^[^ ^179^ ^]^ Researchers have classified DESs as a new class of ILs or IL analogs because they share many physical and chemical properties with ILs.^[^ ^180^ ^]^ The common hydrogen bond acceptors that can form DESs contain quaternary ammonium salt (e.g., choline chloride) and zwitterion (e.g., betaine), whereas thiourea, carboxylic acids (e.g., phenylacetic acid, malic acid, citric acid, and succinic acid), polyols (e.g., ethylene glycol, glycerol, butanediol, and xylitol), amino acids, sugars (e.g., glucose and fructose), and trifluoroacetamide are categorized as common hydrogen bond donors.^[^ ^181^ ^]^

Low‐cost and efficient DESs represented by choline chloride‐glycerol were confirmed to sufficiently degrade the PVDF in a cathode electrode.^[^ ^57^ ^]^ The thermal degradation of PVDF in the DESs was able to separate cathode materials from Al foil. Second, the optimal conditions for cathode material detachment involved a heating temperature of 190 °C, molar ratio of choline chloride to glycerol of 2:1, and heating duration of 10 min, corresponding to a detachment rate of 99.8 wt.%. PVDF has the chemical formula CH_2_‐CF_2_ *_n_* and framework C is tetrahedrally structured. In this context, the F atom of the halogen group located in the second period has a small radius and strong electronegativity, featuring a potent electron‐withdrawing ability and linkage capability. Due to the intense electron‐withdrawing effect of the F atom, the symmetric H atom is highly susceptible to attack, performing an elimination reaction to form an unsaturated double bond. Under the action of DESs at a high temperature, the unsaturated double bonds in PVDF are oxidized to hydroxyl and carbonyl groups, culminating in the formation of unsaturated ketone in the molecular chain. Potassium carbonate‐ethylene glycol DES was also identified for separation of electrode materials.^[^ ^161^ ^]^ Mechanistic analysis showed that the chain reaction of carbonate ions with PVDF caused polymer degradation, which resulted in the effective separation of cathode material particles and Al foil. The electrode materials can be completely separated under mild reaction conditions (100 °C, 20 min), and the DES can be recovered and reused for multiple cycles. The isolated electrode materials are of high purity and have no change in crystal structure.

Compared with systems such as ILs and NMP, DESs stand out in practical application, with the advantages of high security and minimal ecotoxicity, promising to take the lead as new cost‐effective solvents with latent industrial application. However, DESs are applied mainly for recovering metals from the retired power LIBs, and relatively little research has been conducted on their use for the degradation of PVDF.^[^ ^182^, ^183^ ^]^

#### 3.4.4 Bio‐Based Solvents

The global prosperity of the chemical industry is constantly driving the consumption of organic chemical solvents. However, organic solvents are complicated to synthesize, laden with apparent toxicity, and are challenging to access, manage, and dispose of.^[^ ^184^ ^]^ As a result, there has been an uprising of green bio‐based solvents,^[^ ^185^ ^]^ which are derived from raw biomass and/or produced through biochemical methods. Bio‐based solvents are now deemed a new class of environmentally friendly green solvents due to their application advantages, including sustainable regeneration, non‐volatility, non‐toxicity, biodegradability, and recyclability.^[^ ^186^ ^]^

With waste oil as a cheap biomass feedstock and methanol as an ingredient, a bio‐based short‐chain fatty acid methyl ester (FAME) solvent was synthesized.^[^ ^58^ ^]^ At 190 °C, the cathode material of the retired LPF battery was stripped from the Al foil after heating treatment in FAME solvent for 20 min. In this case, FAME represents a transitional‐state solvent capable of dissolving PVDF under heating condition, and the swelling of PVDF in FAME solvent could be attributed to the cross‐linking reaction of hydrogen bonds. In general, FAME solvent offers a new option for recovering PVDF from the retired LIBs. Compared with molten salts, ILs, and DESs, bio‐based solvents deliver environmental and economic benefits and offer a potentially promising direction for future solvent‐based separation and defluorination of PVDF.^[^ ^157^, ^162^ ^]^

#### 3.4.5 Supercritical Fluid Extraction

Supercritical fluid extraction is a novel technique of selective extraction and separation by utilizing a supercritical fluid (i.e., a fluid above the critical temperature and pressure) as an extractant, as this has the characteristics of both liquid and gas by controlling the temperature and pressure.^[^ ^187^, ^188^, ^189^ ^]^ In a common extraction medium, CO_2_ benefits from non‐toxicity, high safety, affordability, and low critical pressure and temperature, and can quickly and reliably separate the target products from the mixture.^[^ ^190^, ^191^, ^192^, ^193^ ^]^ The combination of supercritical carbon dioxide (SC‐CO_2_) and the co‐solvent dimethyl sulfoxide could selectively extract PVDF from the cathode electrode of the retired power LIB.^[^ ^164^ ^]^ In this process, SC‐CO_2_ and dimethyl sulfoxide first achieved mutual solubility, and the flowing SC‐CO_2_ extracted the PVDF molecules dissolved in dimethyl sulfoxide from the pores of the cathode materials. Subsequently, 98.5 wt.% of PVDF was extracted at a temperature of 70 °C, pressure of 80 bar, and duration of 13 min. Fourier Transform Infrared Spectrometer and Thermal Gravimetric Analyzer confirmed that the PVDF surface chemical groups and contents remained intact after extraction, indicating that the recovery of PVDF by SC‐CO_2_—dimethylsulfoxide could be a physical dissolution‐extraction process. Considering the high efficiency, relatively low energy consumption, and environmental friendliness of SC‐CO_2_, this approach has the potential to serve as a new technology for recovering PVDF from the retired LIB cathode electrodes.

A solvent system is a highly targeted and mild separation process for cathode electrodes, i.e., it will only dissolve, swell, or degrade the PVDF in a targeted manner. Existing solvent systems have not been reported to cause damage to metal oxides in the cathode materials or Al foil. In the battery regeneration route, the deactivated PVDF may affect the performance of newly assembled batteries.^[^ ^194^ ^]^ Therefore, pre‐removal is required. To reduce the intervention of impurities, both thermochemistry and solvent chemistry are effective routes.^[^ ^195^ ^]^ Physical crushing‐sorting is more likely to be adopted by the hydrometallurgical route because it may introduce considerable Al and Cu impurities.

Little research has aimed at practical production, the limitations of solution chemistry itself account for this phenomenon: 1) in comparison with the aqueous phase, organic solvents bear certain ecological threats and toxicological properties; 2) high viscosity results in the adhesion of organic solvents on the surface of solid materials, leading to residue problems in practical application; 3) small‐molecule compounds after degradation of PVDF, such as hydrocarbons and organic fluorides, are challenging to separate by traditional techniques when dissolved in organic solvents, resulting in disposal difficulty after solvent saturation. Hence, solvent chemical separation is presently stuck in the laboratory stage and fails to proceed to industrial application.

## 4 Suggestions and Prospects

Vehicle electrification has inexorably become a trend, and the mass production of EVs will lead to a wave of retirement of new EVs globally. However, as the core component of EVs, power LIBs, with their short life cycle and complex manufacturing structure, are likely to pose new challenge of resource recovery. Recycling valuable metals such as Li, Ni, Co, and Mn in the retired LIBs can effectively avoid the risk of upstream raw material scarcity and price fluctuation, producing remarkable environmental and economic benefits. Moreover, the assembled materials of the retired LIBs may trigger metal and/or organic pollution in the recycling process and affect the ecological environment as well as human health through the food chain. In contrast to the recovery of metals, the processing of PVDF—an irreplaceable battery component material—has not been investigated in depth, requiring high priority in the future research.

Pre‐separation of cathode materials and Al foil is a crucial link for the efficiency and quality of metal extraction from cathode materials. The selection of pre‐separation technology depends on the recovery route of metals as the cornerstone for design. However, despite the high priority that ought to be given to PVDF considering its environmental risks and potential hazards, there remains a lack of relevant research on controlling organic fluoride contamination. Consequently, research and development enhancing the corresponding technology is essential for designing appropriate recycling strategies.

In the current industrial recovery of retired power LIBs, thermochemical defluorination tends to be the most prevalent application, with relatively mature technology. However, industrial challenges remain in practice, such as HF corrosion of pipelines, shortened operating life of equipment, and dioxin formation. As such, the focus of subsequent studies should include the research and development of anti‐corrosion materials until a new technological breakthrough is achieved. Moreover, in actual production, the release of fluoride should be strictly controlled through enhanced monitoring, for the purpose of averting secondary environmental contamination arising from dioxin emissions.

Organic fluoride displays the highest ecological risk among the components of retired power LIBs. Despite the contribution of PVDF to the historical development and prevalence of LIBs, endeavors ought to be exerted in future research to eliminate latent environmental pollution at the potential sources. Furthermore, battery research and development companies and scientific research institutions should pay more attention to fluorine‐free alternatives. So far, with the advancement of scientific research, relevant studies on solid‐phase electrolytes have gradually progressed, offering new opportunities for reducing or even abolishing the adoption of organic fluorides.

## 5 Conclusions

The organic binder PVDF in the LIBs binds the electrode materials and current collectors, enhancing the electronic contact among the electrode materials, conductive agents, and current collectors. However, the stable structure of PVDF and the existence of C–F bonds have caused problems in the separation of cathode material and Al foil as well as potential environmental pollution from fluorine‐containing compounds during the recycling process for spent LIBs. We critically reviewed the separation technologies of cathode materials and Al foil as well as the corresponding reaction behavior and transformation mechanisms of PVDF, including physical separation, solid‐phase thermochemistry, solution chemistry, and solvent chemistry. Current awareness of the environmental risks of PVDF during the recycling and disposal of retired LIBs is inadequate, and the research on organic fluorine pollution is insufficient. Strengthening the recycling technology research and developing multi‐technology/inter‐disciplinary collaboration should be the focus of future research. In addition, the development of fluorine‐free alternatives and solid‐state electrolytes can point the way to eliminating fluorine contamination at the source.

## Conflict of Interest

The authors declare no conflict of interest.
